# Nutraceutical preparations in childhood migraine prophylaxis

**DOI:** 10.1186/1129-2377-14-S1-P15

**Published:** 2013-02-21

**Authors:** M Carotenuto, L Antinolfi, MA Faraldo, A Di Dona, M Esposito

**Affiliations:** 1 Department of Child and Adolescent Neuropsychiatry, Italy

## Background

In developmental age migraine is a very common neurological disorder, but very few drugs are disposable. Aim of study is comparing the middle term effect of two nutracetical complexes on frequency, disability grade and intensity of migraine in a paediatric sample.

## Materials and methods

One complex composed by Ginkgolide B/Coenzyme Q10/Riboflavin/Magnesium (complex A) was oral administered as prophylactic therapy twice a day for 6 months to 187 school-aged patients and other one composed by the association of L-triptophan/5hydrossitriptophan (Griffonia simplicifolia)/vitamin PP/vitamina B6 (complex B) to other 187 children with MoA, matched for age (p=0.575) and sex distribution (p=0.918). Each patient kept a journal to record: number and intensity of attacks and concomitant symptoms. To assess the intensity, disability grade and behavioural variations linked to migraine a visual analogue scale (VAS), the PedMIDAS scale and a behavioural scale were administered at the beginning and at the end of treatment.

## Results

Our Results show that the two nutraceutical complexes can reduce all the disabilities aspects of migraine in our samples and the effects of Complex A after six months of treatment seem to have more efficacy than Complex B (Delta% frequency p<0.001, Delta% duration p<0.001, Delta% PedMIDAS p<0.001, Delta% VAS p¡Ü0.001), also for the behavioural aspects (Delta% Behaviour p¡Ü0.001,) that are very important for the dynamics within the family of migraineurs patients, suggesting its improved therapeutic effect in the middle-long term.

## Conclusion

Our findings also suggest that in childhood headache management, the use of alternative treatments could be considered as soft therapy without adverse reactions even in the middle-long term treatment.
